# Characterization of tumor immune microenvironment and cancer therapy for head and neck squamous cell carcinoma through identification of a genomic instability-related lncRNA prognostic signature

**DOI:** 10.3389/fgene.2022.979575

**Published:** 2022-08-29

**Authors:** Lijun Jing, Yabing Du, Denggang Fu

**Affiliations:** ^1^ Department of Neurology, The First Affiliated Hospital of Zhengzhou University, Zhengzhou, China; ^2^ Department of Oncology, The First Affiliated Hospital of Zhengzhou University, Zhengzhou, China; ^3^ School of Medicine, Indiana University, Indianapolis, IN, United States

**Keywords:** head and neck squamous cell carcinoma, genomic instability (GI), long non-coding RNA (IncRNA), tumor immune environment, therapy

## Abstract

Head and neck squamous cell carcinoma (HNSCC) represents one of the most prevalent and malignant tumors of epithelial origins with unfavorable outcomes. Increasing evidence has shown that dysregulated long non-coding RNAs (lncRNAs) correlate with tumorigenesis and genomic instability (GI), while the roles of GI-related lncRNAs in the tumor immune microenvironment (TIME) and predicting cancer therapy are still yet to be clarified. In this study, transcriptome and somatic mutation profiles with clinical parameters were obtained from the TCGA database. Patients were classified into GI-like and genomic stable (GS)-like groups according to the top 25% and bottom 25% cumulative counts of somatic mutations. Differentially expressed lncRNAs (DElncRNAs) between GI- and GS-like groups were identified as GI-related lncRNAs. These lncRNA-related coding genes were enriched in cancer-related KEGG pathways. Patients totaling 499 with clinical information were randomly divided into the training and validation sets. A total of 18 DElncRNAs screened by univariate Cox regression analysis were associated with overall survival (OS) in the training set. A GI-related lncRNA signature that comprised 10 DElncRNAs was generated through least absolute shrinkage and selection operator (Lasso)-Cox regression analysis. Patients in the high-risk group have significantly decreased OS vs. patients in the low-risk group, which was verified in internal validation and entire HNSCC sets. Integrated HNSCC sets from GEO confirmed the notable survival stratification of the signature. The time-dependent receiver operating characteristic curve demonstrated that the signature was reliable. In addition, the signature retained a strong performance of OS prediction for patients with various clinicopathological features. Cell composition analysis showed high anti-tumor immunity in the low-risk group which was evidenced by increased infiltrating CD8^+^ T cells and natural killer cells and reduced cancer-associated fibroblasts, which was convinced by immune signatures analysis via ssGSEA algorithm. T helper/IFNγ signaling, co-stimulatory, and co-inhibitory signatures showed increased expression in the low-risk group. Low-risk patients were predicted to be beneficial to immunotherapy, which was confirmed by patients with progressive disease who had high risk scores vs. complete remission patients. Furthermore, the drugs that might be sensitive to HNSCC were identified. In summary, the novel prognostic GILncRNA signature provided a promising approach for characterizing the TIME and predicting therapeutic strategies for HNSCC patients.

## Introduction

Head and neck squamous cell carcinoma (HNSCC) represents a highly heterogeneous and malignant epithelial-derived tumor occurring in the tongue, oral cavity, nasopharynx, oropharynx, larynx, sinus, salivary gland, and thyroid gland (Siegel et al., 2022). It is estimated that heavy alcohol use, tobacco consumption, and viral infection such as Epstein–Barr virus and human papillomavirus are the main carcinogenic factors (Dhull et al., 2018). The complexity of genetic etiology is the enabling cause of HNSCC tumorigenesis. Current treatment modalities for HNSCC patients include surgery, radiation therapy, chemotherapy, and targeted therapy, while the risk of recurrence is high (Marur and Forastiere, 2016). Early cancer screening for HNSCC is impotent for the population without symptoms (Witek et al., 2017). Most patients are diagnosed at advanced stages which leads to a poor prognosis. Insufficient early diagnostic approaches and deficiency in the clinical use of specific prognostic markers posed an urgent need to identify effective signatures and develop new therapies.

Cell malignant transformation is controlled by many aspects such as genomic instability (GI). GI, including chromosomal instability, microsatellite instability, and epigenetic instability, is characterized by the accumulation of somatic mutations, which are caused by the defects in the process of cell division that may include mutations in DNA damage repair genes or mistakes in DNA replication (Negrini et al., 2010). Although GI increases genetic diversity to accommodate evolution, growing evidence demonstrated that GI acts as one of the major driving determinants during tumorigenesis which occurs in almost human cancers (Yang et al., 2020). Genome-wide profiling has revealed a high burden of genomic mutations in HNSCC that promote substantial inter- and intra-tumoral heterogeneity (Cancer Genome Atlas Network, 2015). Alternations in oncogenic drivers and tumor suppressors are involved in cancer development and treatment response. Carcinogens related to HNSCC such as tobacco exposure, alcohol intake, and ionizing irradiation can accelerate the mutations that lead to DNA repair deficiency and dysfunctional genomic stability pathways. The most frequently amplified regions in HNSCC are on chromosomes 3q, 5p, and 8q (Walter et al., 2013; Cancer Genome Atlas Network, 2015; Hayes et al., 2015). Loss regions are gathered on 3p, 5q, 13q, and 21q. Many driver genes in HNSCC, such as anti-apoptotic kinase protein kinase C (PIK3CA), transcription factors *TP63* and *SOX2*, and telomerase *TERT*, *MYC*, *FHIT*, and *CSMD1*, are located in these areas (Ma et al., 2009). Loss of *FHIT* gene expression is linked to decreased survival in HNSCC (Tai et al., 2004). Mutant *PIK3CA* promotes cell survival and growth by enhancing cyclin D activity and attenuating the apoptotic process (Samuels et al., 2005). Small molecular inhibitors have been developed by targeting patients with wild or mutant *PIK3CA*. Patients with *PIK3CA* mutations showed sensitivity to the mTOR/PI3K inhibitor BEZ-235 (Lui et al., 2013), and patients with wild-type *PIK3CA* were sensitive to PI3K/mTOR inhibitors in combination with MEK inhibitors in preclinical models (Mohan et al., 2015). Thus, GI has been regarded as an evolving hallmark of cancer, and emerging studies have identified its critical role in diagnostic and prognosis implications (Suzuki et al., 2003; Negrini et al., 2010). Aberrant regulation and modification at transcriptomic and epigenetic levels also correlated with GI. The construction of prognostic biomarkers based on the GI signature in HNSCC has been reported (Chen et al., 2021). The tumor microenvironment (TME), especially the tumor immune microenvironment (TIME), attracted rising attention recently because it acts as an important player to shape a unique niche nourishing the malignant properties of tumor cells and affecting response to therapies (Huang et al., 2019). Mutations induced by GI were increased within the TME compared to cells under standard culture conditions, and hypoxic conditions also contributed to mutagenesis (Bindra and Glazer, 2005), suggesting the TME can be an indispensable inducer of GI in cancer cells (Chan et al., 2009; Bizzarri and Cucina, 2014; Sonugur and Akbulut, 2019). It was convinced that hypoxia is a major factor leading to GI, and increased reactive oxygen species can induce single- and double-strand DNA breaks which promote the translocations, deletions, and amplifications in tumor cells (Degtyareva et al., 2013; Helleday and Rudd, 2022). This might indicate the possibility of GI-based signature having implications in reflecting the TME and determining treatment, while current evidence is relatively limited.

Long non-coding RNAs (lncRNAs) represent the transcripts that are longer than 200 nucleotides which do not encode proteins and regulate gene expression at transcriptional, RNA processing, translational, and post-translational levels (Statello et al., 2021), and they were demonstrated to be involved in tumor cell survival, proliferation (Huarte, 2015) and genomic instability (Liu, 2016). Mounting lncRNAs have been identified to promote GI through regulating DNA repair-related gene expression or DNA damage-linked proteins, such as *CUPID1*, *CUPID2,* and *DDSR1* (Polo et al., 2012; Betts et al., 2017). Ritu Chaudhary *et al* found that LINC00460 was abundant in HNSCC tissue and associated with poor patient survival (Chaudhary et al., 2020). Several lncRNA-based signatures showed prognostic effect (Liu G. et al., 2018; Wang et al., 2021) and correlated with TIME landscape (Cao et al., 2022; Li et al., 2022). However, rare studies have reported GI-related lncRNAs potentials in predicting TIME and therapies in HNSCC.

In the present study, we attempted to interrogate transcriptomic profiles and somatic mutation data of patients with HNSCC to develop a GI-associated lncRNAs prognostic signature for characterizing the TIME landscape and predicting therapeutic selection.

## Materials and methods

### Data acquisition and pro-processing

The somatic mutation dataset of 502 patients with HNSCC (VarScan version), the transcriptome profiling based on RNA-seq data (counts), and clinical characteristics was downloaded from the Cancer Genome Atlas (TCGA) database (https://portal.gdc.cancer.gov/). Gene transfer format (GTF) files from the Ensembl database (http://asia.ensembl.org) were employed to convert the Ensembl IDs to gene symbols. mRNA and LncRNAs names were standardized from the HUGO gene nomenclature committee database. A total of 499 patients with complete survival information and somatic mutation were delivered for further analysis. These patients were randomly divided into the training set (*n* = 250) and validation set (*n* = 249) using the “caret” package. The clinicopathological characteristics of the patients in this study were displayed in Supplementary Table S1.

### Identification of genome instability-related lncRNAs

The lncRNA expression profiles of HNSCC patients were extracted and combined with somatic mutation profiles. The cumulative counts of somatic mutations for each patient were calculated. The top 25% of patients within mutation cumulative counts were defined as genomic instability-like (GI, *n* = 127) group, and the bottom 25% of patients were classified as genomic stable-like (GS, *n* = 123) group. The differentially expressed lncRNAs were subsequently determined by comparing the mean expression of lncRNAs between GI- and GS-like groups through the Wilcoxon rank-sum test using limma package (Ritchie et al., 2015). The lncRNAs with Log|Fold Change| > 1.0 and false discovery rate (FDR) < 0.05 were considered as differentially expressed GI-related lncRNAs (GILncRNAs).

### Hierarchical clustering analysis

The expression matrix of lncRNAs was normalized through Z-score analysis. Patients were divided into two clusters by hierarchical clustering analyses using “sparcl,” “pheatmap” and “limma” packages based on the expression of the differentially expressed GILncRNAs. The somatic mutations counts were compared, and the cluster with higher mutations was regarded as a GI cluster, whereas the other was considered as a GS cluster (*p* < 0.05, Mann–Whitney U test).

### Differentially expressed GILncRNA and mRNA co-expression network

To explore the functional mRNA potential co-expressed GILncRNAs, the Pearson correlation analysis was performed based on the lncRNA and mRNA expression levels using the “limma” package. The top 10 mRNAs co-expressed with each GILncRNA were selected according to the Pearson correlation coefficient. The co-expression network of mRNAs and GILncRNAs was illustrated by Cytoscape.

### Functional and pathway enrichment analysis

Functional enrichment analysis of the GILncRNAs co-expressed genes was performed using the clusterProfiler package (Wu et al., 2021) to identify Gene Ontology (GO) term categories, including biological process (BP), cellular competent (CC), molecular function (MF). The pathway referenced from the Kyoto Encyclopedia of Genes and Genomes (KEGG) was also scrutinized. A *p* value <0.05 was considered statistically significant.

### Development and validation of the GInLncRNA-related prognostic signature

The overall survival (OS)-related GILncRNAs were determined using univariate Cox regression analysis in the training set. To avoid overfitting, OS-related GILncRNAs with *p* value < 0.05 were selected by the least absolute shrinkage and selection operator (LASSO) regression with a 10−fold cross−validation using the glmnet package. The GILncRNA-related prognostic signature was constructed using stepwise multivariate Cox stepwise regression analysis. The minimum number of GILncRNAs that comprised of the optimal signature was determined by the Akaike information criterion (AIC) (Vrieze, 2012). The patient’s GILncRNA signature risk score was calculated based on the corresponding GILncRNA signature lncRNA expression levels multiplied by their Cox regression coefficient. The formula for computing GILncRNAs is as follows:

$$\mathrm{GILncRNAs}=\sum_{i}^{n}Coef\text{i}\times A_{i},$$

where “i” represents the signature lncRNA, Coefi is the lncRNA regression coefficient, “A” represents the lncRNA expression value, and “n” represents the number of lncRNAs. Patients with HNSCC were classified into high- and low-risk groups according to the median GILncRNAs score. The prognostic utility of the signature was evaluated by a log-rank test and visualized using the Kaplan–Meier curve. The model discrimination performance was assessed by the receiver operating characteristic (ROC) curve analysis using the timeROC package.

Two external HNSCC datasets GSE41613 (*n* = 97) (Zhao et al., 2022) and GSE42743 (*n* = 103) (Lohavanichbutr et al., 2013) were employed to validate the independently predictive accuracy of GILncRNAs signature. In the GSE41613 set, 21 patients were excluded from this study because they did not succumb to HNSCC. The raw CEL files were downloaded from the GEO database. These two sets (*n* = 179) were normalized and combined following the removal of batch effects by limma and affy packages. Patients were divided into high- and low-risk groups using the same setting.

### Prognostic utility of the GILnRNA signature for patients with different clinicopathological features

To verify whether the GILncRNA signature is a prognostic indicator that is independent of the known clinicopathological features, univariate and multivariate Cox regression analyses were implemented in the training, validation, and whole HNSCC sets. Furthermore, a subgroup analysis was conducted to determine the prognostic efficacy of the signature in the entire HNSCC set with different clinicopathological features including age (≤65 and >65), gender (female and male), tumor grade (G1, G2, and G3–4), tumor stage (I–II and III–IV), pathological N stages (N0 and N1–N3), pathological M stage (M0), and pathological T stages (T1–2, T3, and T4). Patients with each type of clinicopathological parameter were stratified into high- and low-risk groups based on the median risk score. The survival differences between high- and low-risk groups were calculated using the log-rank test and Kaplan–Meier curve.

### Cell composition analysis by multiple immune deconvolution algorithms

To quantify the differences in cell decomposition between patients within high- and low-risk groups, infiltrating cell types in each sample were quantified using multiple immune deconvolution approaches including quantification of the Tumor Immune contexture from human RNA-seq data (quanTIseq) (Finotello et al., 2019); microenvironment cell populations-counter MCPCounter (Becht et al., 2016), TIMER (Li et al., 2016), Cell-type Identification By Estimating Relative Subsets Of RNA Transcripts (CIBERSORT) (Newman et al., 2015), XCell (Aran et al., 2017), and EPI (Racle et al., 2017) based on bulk RNA-seq data. The faction of infiltrating cell types including immune cells between high- and low-risk groups was compared using the Wilcoxon test.

### Characterization of the GILncRNA signature defining the tumor immune microenvironment

The proportions of immune signatures between high- and low-risk patients were quantified by a single sample gene set enrichment analysis (ssGSEA) score. Immune signatures totaling 29 cell types, functions, and pathways, were obtained as described previously (He et al., 2018).

Furthermore, the tumor immune microenvironment was characterized by immune scores and stromal scores which were calculated using the Estimation of STromal and Immune cells in MAlignant Tumor tissues using the Expression data (ESTIMATE) algorithm (Yoshihara et al., 2013). Tumor purity was also assessed based on the ESTIMATE score using a fitted formula as previously described (Yoshihara et al., 2013).

### Immunophenoscore analysis

In recent years witnessed immunotherapy represented by immune checkpoint inhibitors (ICIs) has made remarkable leaps forward in solid tumors. Immunophenoscore (IPS, https://tcia.at/) is an aggregated scoring system based on the expression of the major determinants of tumor immunogenicity including MHC molecules, immunomodulators, effector cells, and suppressor cells (Charoentong et al., 2017) using a random forest approach. The IPS was calculated on a 0–10 scale. IPS was a powerful predictor of response to anti-PD-1 and anti-CTLA-4 antibodies treatment. To compare the responsiveness to ICIs treatment, the IPS levels between high- and low-risk groups were compared using the Wilcoxon test.

In addition, the expressions of programmed cell death protein 1 (PD-1), cytotoxic T-lymphocyte-associated protein 4 (CTLA-4), TIGIT, TIME3, LAG3, CD274, and B7-H4 between high- and low-risk groups were also compared using the Wilcoxon test.

### Prediction of chemotherapeutic response using the GILncRNA signature

Chemotherapy remains the standard treatment for human cancers including HNSCC. To identify the chemicals that are potential responsiveness to patients, the half maximal inhibitory concentration (IC_50_) of each chemical from the GDSC database (Yang et al., 2013) in both groups was predicted and compared using pRRophetic R package (Geeleher et al., 2014).

## Results

### Genomic instability-related lncRNA identification in HNSCC

**FIGURE 1:**
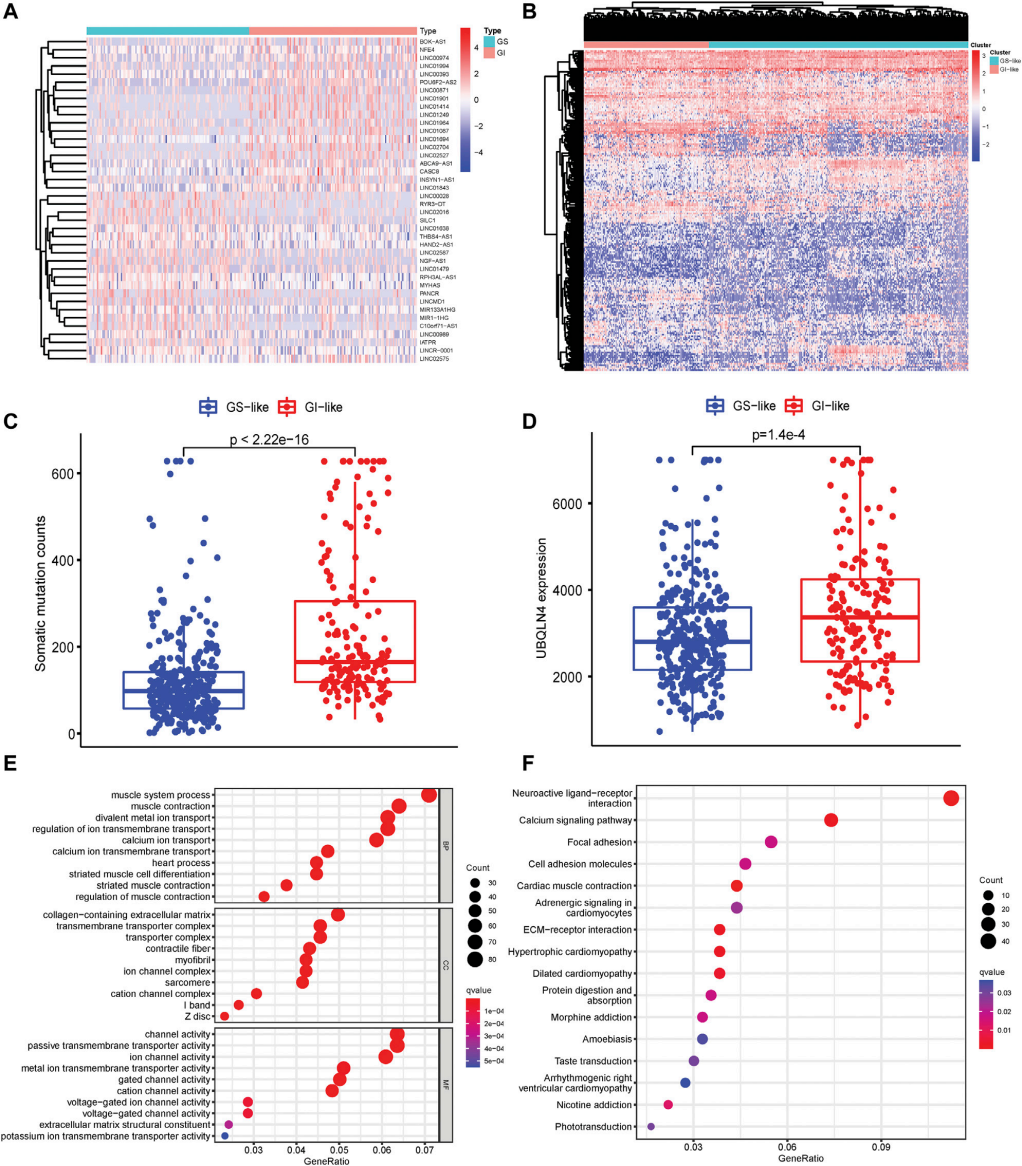
Identification of genomic instability-related lncRNAs in HNSCC. **(A)** Heatmap pf expression of the top 20 differentially expressed lncRNAs in GI-like and GS-like groups. **(B)** Unsupervised clustering of 499 HNSCC patients based on the expression patterns of 199 genomic instability-related lncRNAs. **(C)** Somatic mutation counts in the GI-like and GS-like groups. **(D)** UBQLN4 expression level in the GI-like and GS-like groups. **(E)** GO terms analysis of the differentially expressed lncRNA-related gene coding mRNAs. **(F)** KEGG pathway analysis of the differentially expressed lncRNA-related gene coding mRNAs.

To identify the GI-related lncRNAs in patients with HNSCC, the somatic mutation profile generated by VarScan2 was downloaded and patients were ranked by the cumulative counts of somatic mutations. Patients within the top 25% somatic mutations were divided into genomic instability (GI)-like group, and the bottom 25% somatic mutated patients were classified into genomic stable (GS)-like a group. The differentially expressed lncRNAs were determined between GI- and GS-like groups using the limma package. A total of 67 lncRNAs were found to be down-regulated, while 132 lncRNAs expressions were up-regulated in the GI-like group using |LogFC| > 1 and *p* < 0.05 as the cutoff points (Supplementary Table S2). The top 20 differentially expressed lncRNAs in GI- and GS-like groups were shown in Figure 1A. Unsupervised clustering was performed to assess the underlying molecular physiology of patients in GI- and GS-like groups based on these differentially expressed lncRNAs expression. It revealed high diversity within or between these two groups (Figure 1B), suggesting clustering may not clearly delineate the GI-GS distinction. Accordingly, the frequency of total mutations in the GI-like group was notably higher than that of the GS-like group (Figure 1C). Ubiquilin 4 (*UBQLN4*), a key regulator of DNA damage repair and over-expression in aggressive tumors (Jachimowicz et al., 2019), was significantly increased expression in GI-like vs. GS-like groups (Figure 1D). LncRNAs have been implicated in regulating gene expression at multiple levels such as chromatin structure (Statello et al., 2021). Differentially expressed lncRNA-related coding mRNAs were identified by co-expression network analysis (Supplementary Figure S1). Gene function enrichment analysis indicated that these genes are involved in cell differentiation, transmembrane transport, ion channel activity, and transport activity (Figure 1E). Focal adhesion, ECM-receptor interaction, morphine addiction, and nicotine addiction were the most enriched signaling pathways (Figure 1F), which have been related to cell differentiation, survival, migration, proliferation, and carcinogens during tumorigenesis (Bao et al., 2019).

### Generation of GI-related lncRNA prognostic signature

**FIGURE 2:**
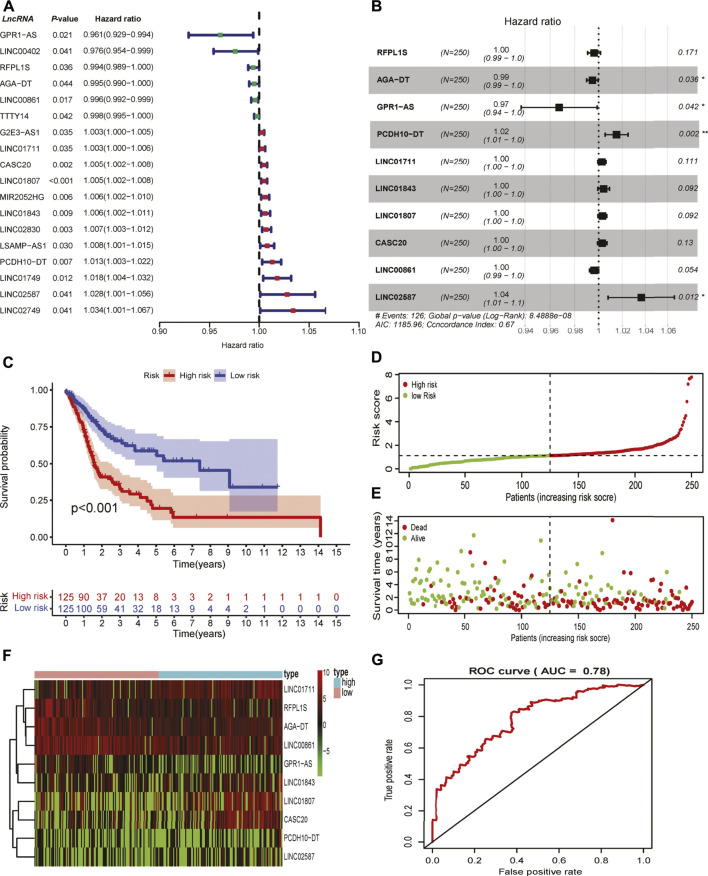
Development of genomic instability-related prognostic signature. **(A)** Identification of overall survival-associated GI-related lncRNAs in the training set in HNSCC. **(B)** The lncRNAs and their coefficients of the prognostic signature were developed by Lasso-Cox regression analysis. **(C)** The Kaplan–Meier curve of patients in high- and low-risk groups in the training set. **(D)** The correlation of the number of patients’ deaths with risk scores. **(E)** The correlation of the number of patients’ overall survival with risk scores. **(F)** The expression of lncRNAs comprised the signature in the high- and low-risk groups in the training set. **(G)** Time-independent receiver operating characteristic curve of the signature in the training set calculated by the area under the curve.

To test the prognostic role of GI-related lncRNAs in HNSCC, 499 patients with clinical survival time were randomly divided into a training set (*n* = 250) and an internal validation set (*n* = 249). Among 199 differentially expressed lncRNAs, 18 lncRNAs were found to be correlated with patients’ OS through univariate Cox regression analysis in the training set (Figure 2A). Elevated expression of four lncRNAs (LINC00402, RFPL1S, LINC00861, and TTTY14) and reduced LINC02587 in the GS-like group were predicted to be protective factors, while decreased GPR1-AS and AGA-DT expression were risk factors for HNSCC patients. The remaining lncRNAs were up-regulated in GI-like groups that were associated with unfavorable survival (Figure 2A). Lasso-Cox regression analyses were conducted to screen independent prognostic lncRNAs that were used to develop the GI-related lncRNAs signature. As shown in Figure 2B, 10 survival-related lncRNAs constituted the final GI-related lncRNA signature (GILncRNAs) as follows:

GILncRNAs = RFPL1S * (−0.0038) + AGA-DT * (−0.0052) + GPR1-AS * (−0.0331) + PCDH10-DT * (0.0151) + LINC01711 * (0.0027) + LINC0183 * (0.0042) + LINC01807 * (0.0032) + CASC20 * (0.0030) + LINC00861 * (-0.0038) + LINC02587 * (0.0359).

The risk score for the individual patient in the training set was calculated, and patients were divided into low- and high-risk groups using the median risk score as the cutoff value. Kaplan–Meier curve analysis showed that patients with high risk scores were reduced overall survival as compared to those patients with low risk scores (*p* < 0.001, Figure 2C). The number of deaths increased along with risk score rises (Figures 2D,E). These lncRNAs that comprised the signature showed differential expression in high- and low-risk groups (Figure 2F). The robustness of the GILncRNAs signature was evaluated using a time-dependent ROC curve. The area under the curve (AUC) of the signature was 0.73 in the training set (Figure 2G), indicating the predictive performance was satisfactory.

### Validation of the GILncRNA signature in validation sets

**FIGURE 3:**
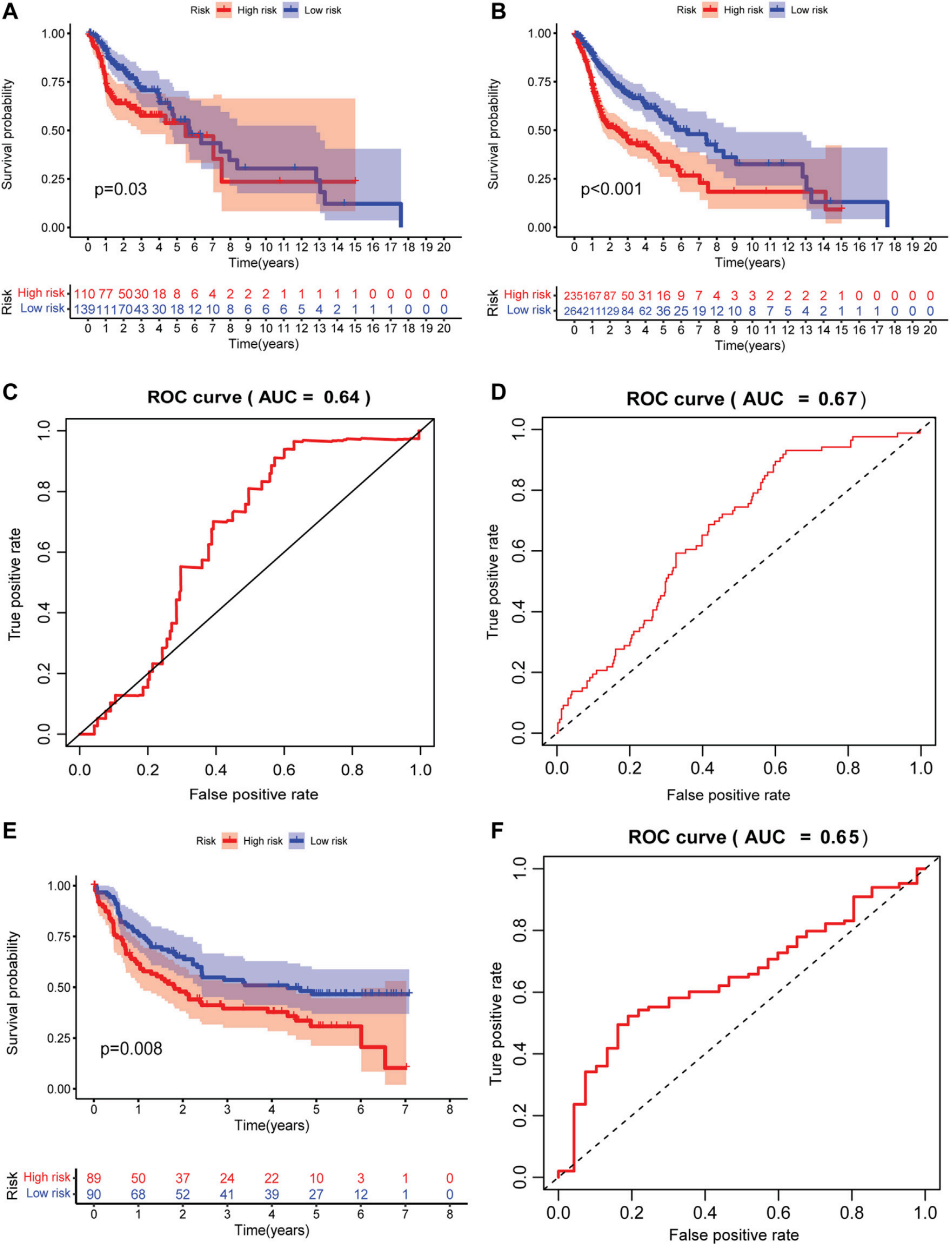
Internal and external validation of the prognostic signature. **(A)** The Kaplan–Meier curve of patients in high- and low-risk groups in the internal validation set. **(B)** The Kaplan–Meier curve of patients in high- and low-risk groups in the entire HNSCC validation set. **(C)** Time-independent receiver operating characteristic curve of the signature in the internal validation set calculated by the area under the curve. **(D)** The time-independent receiver operating characteristic curve of the signature in the entire HNSCC validation set is calculated by the area under a curve. **(E)** The Kaplan–Meier curve of patients in high- and low-risk groups in the external HNSCC validation set (GSE41613 and GSE42743). **(F)** The time-independent receiver operating characteristic curve of the signature in the external HNSCC validation set (GSE41613 and GSE42743) is calculated by the area under the curve.

Since the signature was established based on the limited number of patients, internal validation set and external independent HNSCC sets (GSE41613 and GSE42743) from the GEO database were used to verify the predictive capability of the GILncRNAs signature. Patients were classified into high- and low-risk groups using the same scheme as that in the training set. In the validation set, decreased OS was observed in patients with high risk scores in contrast to those who have low risk scores (*p* = 0.03, Figure 3A). A markedly shorter OS was seen in the entire HNSCC combined with the training and validation sets (*p* < 0.001, Figure 3B). The AUCs of the signature in the validation set and entire HNSCC set was 0.64 (Figure 3C) and 0.67 (Figure 3D), respectively, suggesting it has a moderate capacity for monitoring prognosis.

To test the predictive accuracy of GILncRNAs signature in independent external datasets, patients in high-risk group have better OS as compared to those in the low-risk group (*p* = 0.008, Figure 3E). The AUC of the signature in this validation set was 0.65 (Figure 3F), implying that the signature also has medium performance in predicting patients’ OS in an array-based platform.

### Prognostic utility of the GILncRNA signature for patients with different clinicopathological features

It has been widely known that tumor progression has tightly correlated with patients’ clinical features. Clinical stratification analysis was implemented including age (>65 and ≤65), gender, pathological tumor node metastases system (TMN), tumor stage (I–II and III–IV), and grade (I, II, and III–IV). We found that patients with high risk scores had an unfavorable OS rate than patients with low risk scores in these clinicopathological features, suggesting the GILncRNAs signature was a reliable prognostic indicator (Supplementary Figure S2A–O).

### Regulation of N6-methyladenosine (m6A) messenger RNA methylation regulators and oncogenic drivers by the GILncRNA signature

Dysfunction of m6A mRNA methylation regulators was involved in mediating lncRNAs metabolism and cancer progression (Wang T. et al., 2020). Increasing reports have demonstrated the crucial functions of m6A regulators in impairing the CD8^+^ T cell anti-tumor effect and increasing the resistance to anti-PD-1 therapy (Zhang et al., 2020; Guo et al., 2021). The m6A writer RBM15, and readers (*YTHDC2*, *YTHDF2*, *YTHDC1*, *YTHDF1*) were up-regulated in patients with low-risk scores, while reduced eraser *FTO* expression was observed in the high-risk group (Supplementary Figure S3A). For instance, *FTO* plays an oncogenic role in lung squamous cell carcinoma by decreasing m6A levels and mRNA stability of *MZF1* (Liu J. et al., 2018). What’s more, the somatic mutation profiles were assessed and plotted (Supplementary Figure S3B). The frequencies of the oncogenic drivers including tumor suppressor 53 (*TP53*) and *CDKN2A* mutations were increased in high-risk patients (Supplementary Figure S3C, D).

### Cell composition analysis by multiple immune deconvolution algorithms

**FIGURE 4:**
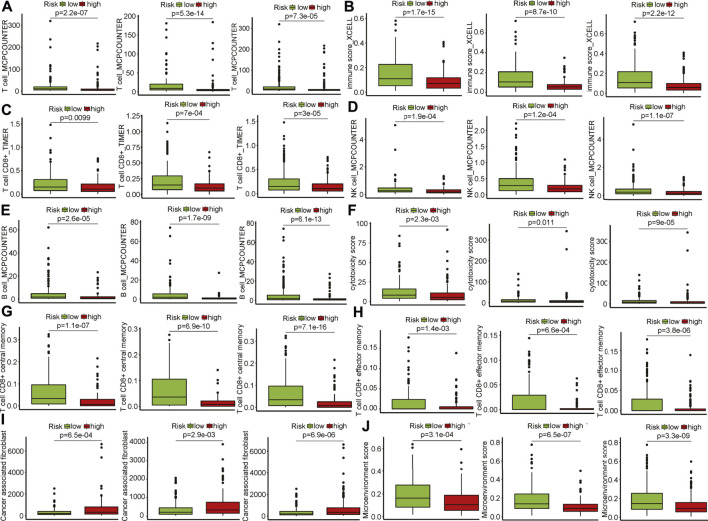
Infiltrating cell type analysis using multiple deconvolution algorithms. **(A)** Total T cells infiltrated in patients of the high- and low-risk groups in training and validation sets (left to right: training, internal, entire HNSCC sets). **(B)** Immune score of patients in the high- and low-risk groups in training and validation sets (left to right: training, internal, entire HNSCC sets). **(C)** CD8^+^ T cells infiltrated in patients of the high- and low-risk groups in training and validation sets (left to right: training, internal, entire HNSCC sets). **(D)** Natural killer cells infiltrated in patients of the high- and low-risk groups in training and validation sets (left to right: training, internal, entire HNSCC sets). **(E)** B cells infiltrated in patients of the high- and low-risk groups in training and validation sets. (left to right: training, internal, entire HNSCC sets). **(F)** Cytotoxicity scores of patients in the high- and low-risk groups in training and validation sets (left to right: training, internal, entire HNSCC sets). **(G)** Central memory CD8^+^ T cells infiltrated in patients of the high- and low-risk groups in training and validation sets (left to right: training, internal, entire HNSCC sets). **(H)** Effector memory CD8^+^ T cells infiltrated in patients of the high- and low-risk groups in training and validation sets (left to right: training, internal, entire HNSCC sets). **(I)** Cancerassociated fibroblasts infiltrated in patients of the high- and low-risk groups in training and validation sets (left to right: training, internal, entire HNSCC sets). **(J)** Microenvironment scores of patients in the high- and low-risk groups in training and validation sets (left to right: training, internal, entire HNSCC sets).

Cell composition that infiltrated patients matters in anti-tumor immunity. Multiple deconvolution algorithms were used to quantify the infiltrating various cells in the TME of HNSCC patients. The fractions of different infiltrating cells between high- and low-risk groups were compared in the training (Supplementary Figure S4A), internal validation set (Supplementary Figure S4B), and entire TCGA set (Supplementary Figure S4C). Among these infiltrating immune cells, we found that the fraction of total T cells were elevated in patients within the low-risk group in the training and validation sets that calculated using MCPCounter (Figure 4A), suggesting enhanced anti-tumor activities in this group. This was evidenced by a raising immune score (Figure 4B). The frequency of CD8^+^ T, natural killer (NK), and B cells was also notably increased in the patients with low-risk scores across the training and validation sets (Figures 4C–E). CD8^+^ T and NK cells were the main players in killing tumors, and the higher cytotoxicity score convinced this hypothesis (Figure 4F). Moreover, central/effector memory CD8^+^ T cells were supposed to be functional tumor-reactive T cells for anti-tumor immunotherapies (Klebanoff et al., 2005) and were observed to be higher in the low-risk group (Figures 4G,H). Cancer-associated fibroblasts (CAFs) are considered one of the most abundant and key factors that have diverse functions in the TME (Sahai et al., 2020). Six fibroblast markers (Costa et al., 2018) [fibroblast activation protein (*FAP*), integrin b1 (*ITGB1*), a-smooth muscle actin (*aSMA*), fibroblast-specific protein-1 (*FSP-1*), platelet-derived growth factor receptor b (PDGFRB), and caveolin-1 (*CAV1*)] have been used to delineate CAFs subtypes which were linked to immunosuppression and resistance to immunotherapy in breast cancer (Kieffer et al., 2020). Elevated infiltrating CAFs were seen in patients with high risk scores (Figure 4I), indicating that CAFs might play a cancer-promoting role in tumorigenesis. To further investigate the heterogeneity of CAFs defined by the signature, we found that *FDGFRB, FAP, CAV1, aSMA,* and *ITGB1* were increased expression in patients with high risk scores, while *FSP-1* was down-regulated (Supplementary Figure S4D). Patients were classified into three subtypes (Cluster A, B, and C) based on the expression of these markers using the ConsensusCluster package (Seiler et al., 2010) (Supplementary Figure S4E). Patients in Cluster C had better survival as compared to patients in clusters A and B (Supplementary Figure S4F). Furthermore, Cluster C has lower risk scores vs. Cluster A and B (Supplementary Figure S4G)*.* These data suggested high-risk patients have the suppressive TIME as compared to patients in low-risk group (Supplementary Figure S4J). In summary, the GILncRNAs signature was an indicator of the TIME landscape.

### Characterization of the GILncRNA signature-related tumor immune microenvironment

**FIGURE 5:**
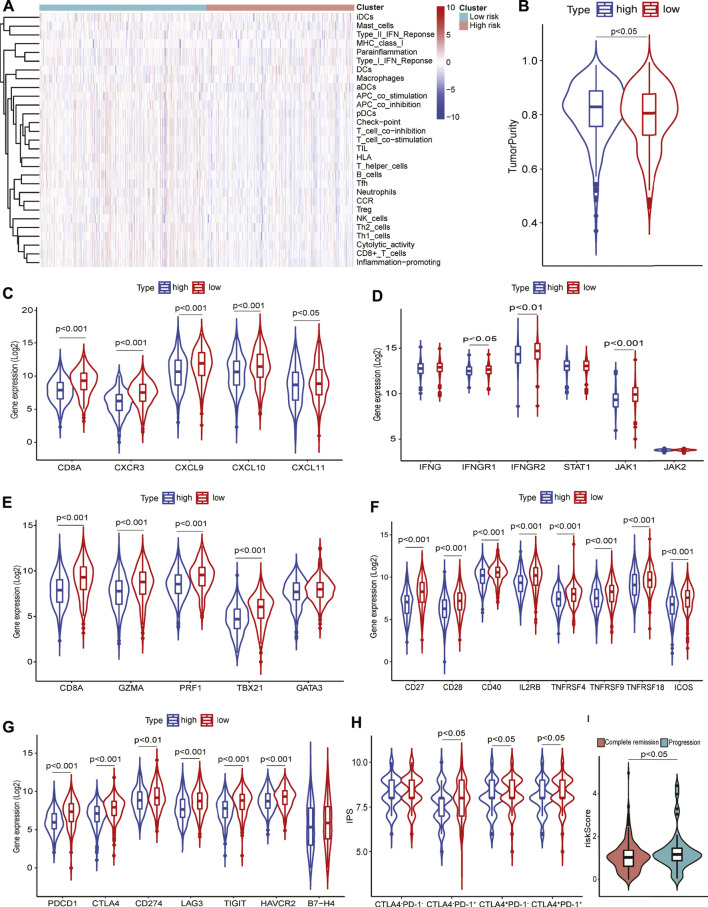
Tumor immune microenvironment analysis using 29 immune signatures through ssGSEA. **(A)** The immune signatures between high- and low-risk groups. **(B)** Tumor purity in high-and low-risk groups. **(C)** T cell response signatures expression in high- and low-risk groups. **(D)** T helper/IFNγ signatures expression in high- and low-risk groups. **(E)** Cytotoxic signatures expression in high- and low-risk groups. **(F)** Co-stimulatory signatures expression in high- and low-risk groups. **(G)** Co-inhibitory signatures expression in high- and low-risk groups. **(H)** Immunophenoscore levels in high- and low-risk groups. **(I)** Risk scores in patients with complete remission or progressive disease.

To further characterize the TIME defined by the GILncRNA signature, the proportions of 29 immune signatures in patients between the high- and low-risk groups were quantified by ssGSEA score. We found that increased infiltrated immune cells and enhanced functional immune-associated signatures in the low-risk group as compared to the high-risk group (Figure 5A), such as CD8^+^ T cells, APC-related co-stimulatory and co-inhibitory signals, tumor-infiltrating lymphocytes (TILs), checkpoint inhibitors, and cytolytic activity, suggesting patients in the low-risk group have hot immune reactive activities in anti-tumor immunity. Patients in the low-risk group showed increased immune scores and microenvironment scores estimated using the ESTIMATE algorithm (Supplementary Figure S4H), while higher tumor purity was found in the high-risk group in contrast to that in the low-risk group (Figure 5B). Previous studies showed that *CXCR3*-expressing activated T cells were involved in the growing recruitment of infiltrating effector T cells in the TME through interaction with its receptors *CXCL9*, *CXCL10*, and *CXCL11* (Groom and Luster, 2011). We found that *CXCR3* and its receptors significantly increased expression in patients within the low-risk group (Figure 5C). It has been demonstrated that increased IFNγ-expressing CD8^+^ T cells that infiltrated the TME are an important marker of the responsiveness to immune checkpoint inhibitors (ICIs) based immunotherapies and can also promote to up-regulation of PD-1/PD-L1 expression (Karachaliou et al., 2018). This was confirmed that the expression of T helper/IFNγ signatures including *IFNG*, *IFNGR1*, *IFNGR2*, *STAT1*, *JAK1*, and *JAK2* were markedly increased in the patients within the low-risk group as compared to the patients within the high-risk group (Figure 5D). This was consistent with the evidence that more *IFNγ* release can induce apoptosis of lung cancer cells through activating the JAK-STAT1 pathway (Song et al., 2019). In addition, T helper cell response signatures such as *CD8A*, *GZMA*, *TBX21*, *GATA3*, and *PRF1* were elevated expression in the low-risk group (Figure 5E). Down-regulation of co-stimulatory (Figure 5F) and co-inhibitory (Figure 5G) immune modulator expression was seen in patients with high risk scores which contributes to confirming the truth of the reduced cytotoxic phenotype of T cells, particularly CD8^+^ T cells, in the TME.

### Immunophenoscore analysis

Increased expression of immune checkpoint molecules such as *PD-1*, and *CTLA-4* in the low-risk group (Figure 5G) prompted us to investigate the patients’ response to ICIs therapy. Patients in the low-risk group had higher IPS levels than that in the high-risk group according to anti-PD-1 and/or anti-CTLA-4 therapies (Figure 5H). Furthermore, interrogation of the predictive potential of GILncRNAs signature for patients receiving clinical treatments indicated that the risk scores were significantly higher in the disease progressive patients than in complete remission patients (Figure 5I), showing its capacity in predicting treatment response for HNSCC.

### Prediction of chemotherapeutic response by the GILncRNA signature

**FIGURE 6:**
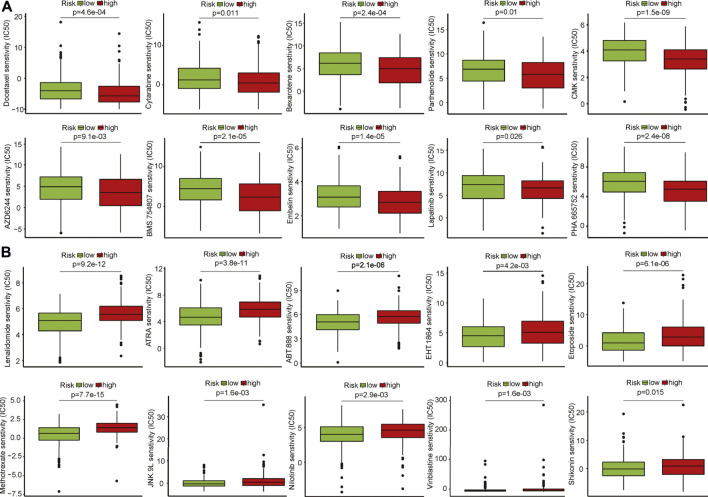
Identification of potential therapeutic drug response to HNSCC patients. **(A)** Drugs that are potentially sensitive to the patients in the high-risk group. **(B)** Drugs that are potentially sensitive to the patients in the low-risk group.

To explore the potential responsiveness of patients to chemicals/drugs that might be used to treat HNSCC based on the IC_50_ data. We noted that 40 chemicals were predicted to have low IC_50_ patients in the high-risk group compared to those in the low-risk group, suggesting patients were more sensitive to these chemicals/drugs (Figure 6A). Meanwhile, 18 chemicals showed higher IC_50_ in patients within the high-risk group, meaning they might be potentially effective for treating HNSCC (Figure 6B). Further verification of these identified chemicals in anti-HNSCC *in vitro* and *in vivo* is warranted.

## Discussion

Head and neck cancer (HNC) is one of the most death-causing malignancies that arise from the lip, oral cavity, nasopharynx, oropharynx, tongue, and larynx tissues (Sung et al., 2021). HNC is mainly comprised of HNSCC, accounting for over 90% of patients. Most patients were diagnosed at advanced or metastatic stages leading to poor outcomes (Siegel et al., 2022). Emerging therapies including molecular targeted therapy, immunotherapy, and combination therapy with standard treatments improved patients’ life quality and extended survival. The small fraction of patients that show responsiveness to immunotherapy limited its benefit in most cases owing to the suppressive tumor microenvironment (Economopoulou et al., 2016). HNSCC is understood to be synergistic and driven by the mutations in many oncogenes and tumor suppressor genes (Carson et al., 2011). GI is the hallmark of HNSCC and the inducer of these mutations, indicating that GI plays a crucial role in mediating TME. However, there are rare consistent prognostic biomarkers for HNSCC due to high heterogeneous genomics and complex etiology (Beck and Golemis, 2016; Yang et al., 2020). Studies have revealed that lncRNAs hold potential in the pathogenesis, diagnosis, prognosis, and targeted treatment of patients with HNSCC by promoting DNA damage and regulating the cell cycle (Guglas et al., 2017; Wang Y. et al., 2020; Jiang et al., 2022; Li et al., 2022), while the role of GI-associated with lncRNAs in the prediction of TIME and therapeutics for HNSCC has not been systematically assessed.

In this study, we profiled genome-wide somatic mutations of HNSCC from the TCGA database and identified the top 25% and bottom 25% number of mutations as GI- and GS-like groups. GI-related lncRNAs were determined by differentially expressed lncRNAs. Correlation analysis identified lncRNA-related protein-coding genes, and these genes were tightly enriched in several KEGG pathways that are involved in tumorogenesis and disease progression (Bao et al., 2019). Since previous studies have demonstrated that these lncRNAs were involved in the tumorigenesis of various cancers, this might indicate that they are key mediators in the pathogenesis of HNSCC. Among the lncRNAs included in the signature, LncRNA LINC01711 was demonstrated to promote the occurrence and development of esophageal squamous cell carcinoma through increasing cell proliferation, migration, and invasion by the miR-326/FSCN1 axis (Xu et al., 2021). Elevated LINC01711 expression in bladder cancer was found to be associated with decreased survival (Du et al., 2021). In addition, LINC01711 expression was positively correlated with TGF-β1, a key factor in the TGF-β signaling pathway (Lee et al., 2005). These data suggested that LINC01711 might play a tumor-promoting role in HNSCC development. lncRNA cancer susceptibility 20 (CASC20) was reported to serve as a tumor promoter by promoting the metastasis of human gastric cancer cells by the miR-143-5p/MEMO1 molecular axis (Shan et al., 2022). LINC01843 increased expression in patients and correlated with poor survival in lung adenocarcinoma (Li et al., 2020; Zheng et al., 2021) and colon cancer (Zhou et al., 2018). LINC00861 had an inhibitory function in cervical cancer cells by regulating PTEN/AKT/mTOR signaling pathway (Liu et al., 2021). The remaining lncRNAs functions in HNSCC need further clarification. Thus, we developed 10 survival-associated GI-related lncRNAs constituted prognostic signature that has robust survival stratification capacity in the training set using LASSO-Cox regression analysis, which was validated in the external and entire TCGA HNSCC sets. In addition, the signature has emphasized the evident applicability of predictive utility in the external set combined from two HNSCC cohorts. This suggested that the GILncRNAs signature showed as a superior indicator for monitoring patients’ survival. The calculation of predictive performance showed its reliable and stability in RNA-seq and array-based platforms. Yun Chen et al reported a genomic instability associated with lncRNA prognostic signature that shows potential for survival prediction of patients with HNSCC, while it was not validated in an external HNSCC set, and the applicability of the signature was not investigated (Chen et al., 2021). Our signature retained the comparable performance and holds superior capability to reflect the TIME. Clinical stratification analysis further validated the prognostic value of GILncRNAs signature in patients with different characteristics. These data suggested that the signature was potentially implicated in clinical practice, whereas validation in multi-center derived HNSCC cohorts is required.

Studies have shown that TME is of the major players in tumor progression and inducers of genomic instability in tumor cells (Sonugur and Akbulut, 2019). Growing evidence indicated that oncogenesis is characterized by defects in the immune system as tumor cells could evade immunosurveillance resulting from the accumulation of genetic mutations and cancer heterogeneity (Economopoulou et al., 2016). Immunotherapy including ICIs targeting *PD-1/PD-L1* and *CTLA-4* in HNSCC has shown as potential therapeutics in HNSCC, while TIME affects the responsiveness and resistance of treatment. Infiltrated cell types in the TME between high- and low-risk groups were detected by multiple deconvolution algorithms. We found that cytotoxic elements such as CD8^+^ T cells and NK cells were notably increased in patients with low-risk scores. Increasing infiltration of total T cells, especially CD8^+^ T cells acted as the main killer of anti-tumor immunity of solid tumors (Lanitis et al., 2017). *CXCR3*-expressing activated T cells played an important role in the recruitment of effector T cells, and we found that its receptors and ligands were up-regulated in low-risk patients. This was convinced that high infiltrated central/effector CD8^+^ T cells in patients with low-risk scores. Emerging concepts show that co-stimulatory and co-inhibitory molecules have a pivotal role in T-cell activation, differentiation, and effector function (Chen and Flies, 2013). The expression of these inhibitory and inhibitory molecules such as *CD40*, *ICOS*, *PD-1*, *CTLA-4*, *PD-L1*, and *TIGIT* was elevated in the patients within the low-risk group. This might mean increased T-cell tumor-reactive activation in the low-risk patients. Immunotherapy targeting *PD-1/PD-L1* and *CTLA-4* may be effective for this subset of patients stratified by the GILncRNA signature. Patients with low-risk scores showing high IPS levels confirmed the notion. We did observe high risk scores in patients with the stable disease compared to CR patients. Recent studies have shown that CAFs are associated with anti-PD-1 immune checkpoint inhibitors treatment (Costa et al., 2018; Kieffer et al., 2020). CAFs subpopulations have diverse functions in TIME and modulating response to treatments in HNSCC patients (Obradovic et al., 2022). We found that CAFs were highly infiltrated in patients with high risk scores. To investigate the heterogeneity of CAFs in patients defined by the signature, patients were classified into three subtypes (Cluster A, B, and C), and Cluster C with decreased survival had higher risk scores as compared to the other two subtypes. Among the signatures that marked CAFs, the expression of five markers (FAP, ITGB1, aSMA, PDGFRB, and CAV1) was elevated in patients with risk scores. These data supported that the signature could characterize the TIME status and predict cancer therapies.

m6A is one of the most prevalent drivers in modifying the mRNAs and lncRNAs by affecting RNA metabolism (Yue et al., 2015). Several m6A regulators were differentially expressed between the high- and low-risk group including writers, erasers, and readers. Decreased methyltransferases *METTL14* can promote the malignant attribute of glioblastoma stem cells while suppressing the demethylase *FTO* plays the opposite role (Cui et al., 2017). Reduced *FTO* expression and increased *METTL14* expression were observed in the low-risk group, suggesting m6A might be involved in regulating lncRNAs in both groups. In addition, the frequency of oncogenic drivers such as *TP53* and *CDKN2A* was higher in the high-risk group, which might be a contributor to progressive tumors. Identification of novel therapies is still an urgent need for patients with HNSCC. We found that some chemicals that might be sensitive to patients in a high- or low-risk group based on expression-based prediction. Some of these drugs have been used in treating cancers including HNSCC. Docetaxel/paclitaxel-containing schemas showed as a promising beneficial therapy for recurrent and/or metastatic HNSCC (Catimel et al., 1994; Shin and Lippman, 1999), and low IC_50_ of docetaxel was seen in high-risk patients. Parthenolide was reported to treat oral cancer cells by inducing apoptosis (Yu et al., 2015), and we found that it is sensitive to high-risk patients. Bexarotene might be effective in patients with high risk scores, and its anti-HNSCC efficacy was evidenced by targeting the PPARγ/RXRα heterodimer oral cancer preclinical test (Rosas et al., 2022). Further validation of these identified drugs could consolidate the findings.

There are some limitations in our study that need to be cautious when interpreting the results. Although the robust GILncRNAs signature was generated and validated based on the retrospective datasets, it still needs to be validated in multiple sets, particularly in a clinical setting. The underlying mechanisms of the difference in TME and drug effectiveness predicted by the signature require further *in vitro* and *in vivo* studies.

## Conclusion

In conclusion, a reliable genomic instability-related lncRNA prognostic signature was developed and validated for patient survival stratification using RNA-seq and array-based datasets. The TIME landscape of HNSCC patients in low- and high-risk groups was characterized by relatively comprehensive approaches, and the signature also provided the feasibility of the potential responsiveness to targeted immunotherapy. Several drugs that were sensitive to patients with HNSCC were identified. Our findings provided a robust prognostic signature and helped gain a deeper understanding of the TIME landscape for HNSCC, which could facilitate the development of novel cancer therapeutics.

## Data Availability

The original contributions presented in the study are included in the article/Supplementary Material; further inquiries can be directed to the corresponding author.

## Abbreviations

AUC, area under the curve; CAFs, cancer-associated fibroblasts; CIBERSORT, Cell-type Identification By Estimating Relative Subsets Of RNA Transcripts; CTLA-4, cytotoxic T-lymphocyte-associated protein 4; ESTIMATE, Estimation of STromal and Immune cells in MAlignant Tumor tissues using Expression data; GDSC, Genomics of Drug Sensitivity in Cancer; GEO, Gene Expression Omnibus; GO, Gene Ontology; GTF, gene transfer format; HNSCC, head and neck squamous cell carcinoma; IPS, immunophenoscore; KEGG, Kyoto Encyclopedia of Genes and Genomes; LASSO, least absolute shrinkage, and selection operator; MCP-counter, microenvironment cell populations-counter; OS, overall survival; PD-1, programmed cell death 1; quanTIseq, quantification of the Tumor Immune contexture from human RNA-seq data; ROC, receiver operating characteristic; ssGSEA, single-sample gene set enrichment analysis; TCGA, The Cancer Genome Atlas; ICIs, immune checkpoint inhibitors; TILs, tumor-infiltrating lymphocytes; TIME, tumor immune microenvironment; TME, tumor microenvironment.
