# Supplementary figures and images for: Characterization of tumor immune microenvironment and cancer therapy for head and neck squamous cell carcinoma through identification of a genomic instability-related lncRNA prognostic signature
Source: Front Genet. 2022 Aug 29;13:979575. doi: 10.3389/fgene.2022.979575 (PMC9465021; doi:10.3389/fgene.2022.979575)

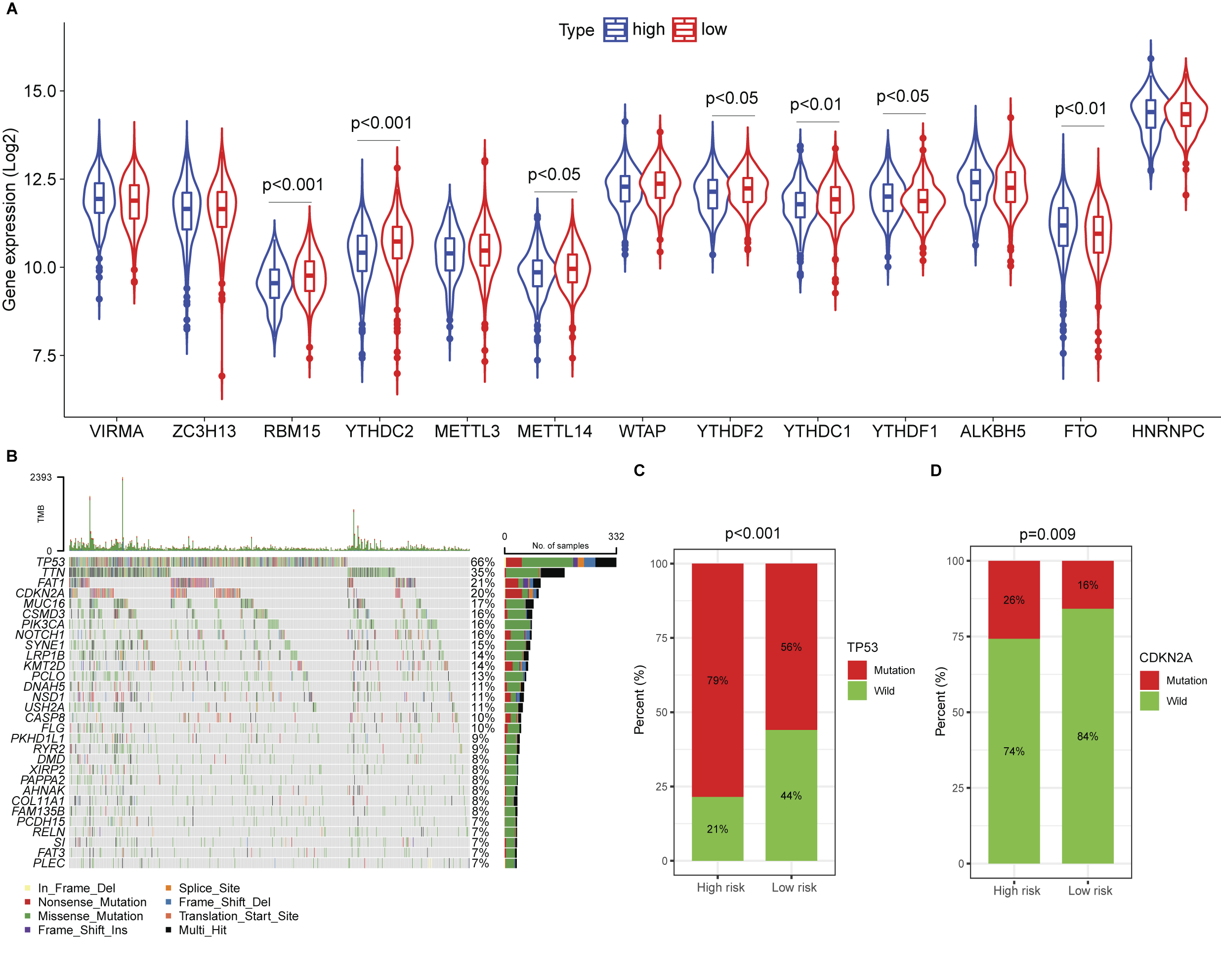

Supplement: Supplementary file 3 [file Image3.tif]

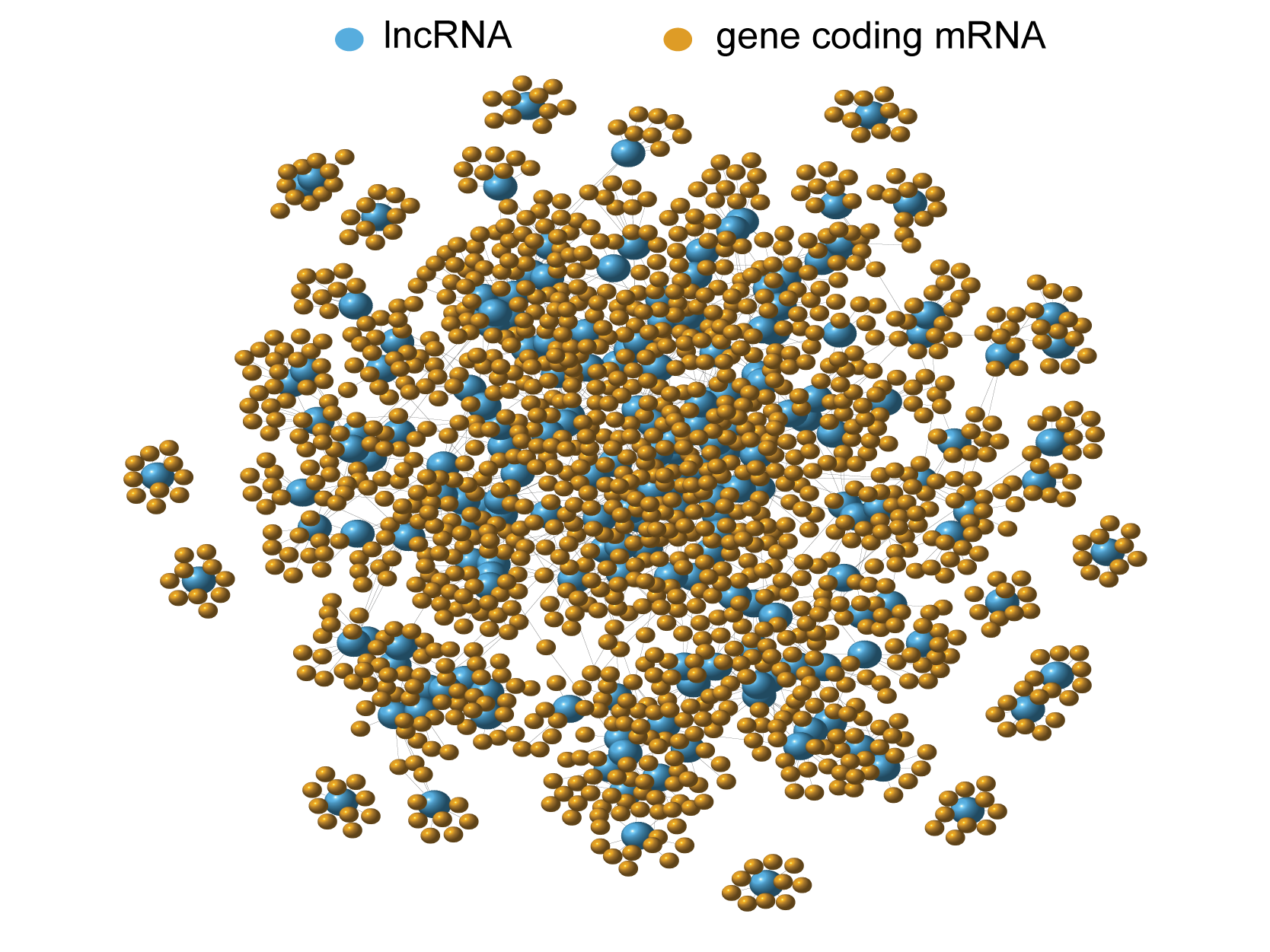

Supplement: Supplementary file 4 [file Image1.TIF]
